# First demonstration of protective effects of purified mushroom polysaccharide-peptides against fatty liver injury and the mechanisms involved

**DOI:** 10.1038/s41598-019-49925-0

**Published:** 2019-09-23

**Authors:** Shuang Zhao, Shuman Zhang, Weiwei Zhang, Yi Gao, Chengbo Rong, Hexiang Wang, Yu Liu, Jack Ho Wong, Tzibun Ng

**Affiliations:** 1 Institute of Plant and Environment Protection, Beijing Academy of Agriculture and Forestry Sciences, Beijing, 100097 China; 2 Beijing Key Laboratory of Fruits and Vegetable Storage and Processing, Key Laboratory of Vegetable Postharvest Processing, Ministry of Agriculture, Beijing, 100097 China; 3 Institute of Medicinal Plant Development, Chinese Academy of Medical Sciences & Peking Union Medical College, Beijing, 100193 China; 4 Beijing Xicheng District Health Care Center for Mothers and Children, Beijing, 100053 China; 5 State Key Laboratory for Agrobiotechnology and Department of Microbiology, China Agricultural University, Beijing, 100193 China; 6 School of Biomedical Sciences, Faculty of Medicine, The Chinese University of Hong Kong, Shatin, New Territories, Hong Kong, China

**Keywords:** Chromatography, Fungal biology, Nutrition

## Abstract

Fatty liver (FLD) disease is a consequence of metabolic syndrome, which is a health problem worldwide with a phenomenal rise in prevalence. In this study, two hepatoprotective polysaccharide-peptides were extracted from the mushroom *Auricularia polytricha* followed by chromatographic fractionation of the extract on the ion exchanger DEAE-cellulose and gel filtration on Sephadex-200 to yield two purified fractions: APPI and APPII. The monosaccharide compositions, FT-IR, N-terminal sequences, internal peptide sequences and molecular weights of the two fractions were determined. Furthermore, their hepatoprotective effect on human hepatoma HepG2 cells *in vitro* and in an animal model of fatty liver disease was evidenced by the findings that APPI and APPII diminished lipid deposit in cells, blood and the liver, increased cellular antioxidant activity and viability, and protected the liver against injury. The mechanistic study revealed that APPI and APPII activated the adiponectin pathway, up-regulated expression of genes controlling free fatty acid (FFA) oxidation, such as *AMPK*, *CPTl*, *ACOX1* and *PPARα* genes, enhanced lipid metabolism, preserved hepatic function, promoted the antioxidant defense system and reduced lipid peroxidation. Hence the bioactive compounds of *A*. *polytricha* could serve as therapeutic agents in the food and pharmaceutical industries.

## Introduction

The liver is an important organ in charge of the metabolism of lipids, glucose, proteins, alcohol, drugs, and chemicals^1^. Accumulating data suggest that excessive intake of alcohol and fat induces improper triglyceride metabolism in the hepatocytes^2^. When synthesis proceeds faster than anabolism, triglycerides accumulate in the liver. The excessive lipid in the hepatocytes, exceeding 5–10% of the liver weight, is the main pathogenetic factor of fatty liver disease (FLD)^3,4^. The multiplicity of etiologic factors of FLD, include genetic, dietary factors, insulin resistance, and adipokines^5^. FLD is a metabolic syndrome, which has become a vital health issue with a phenomenal escalation in prevalence. Fatty liver disease comprises alcoholic fatty liver disease and nonalcoholic fatty liver disease (NAFLD). Alcohol-related liver disease and NAFLD are common chronic hepatic derangements^6^. The disease affects grown-ups and children alike and is getting more and more rampant in industrialized as well as developing countries. Cirrhosis associated with NAFLD is likely to be a priority for hepatic transplantation in the foreseeable future. The incidence of liver cancer associated with NAFLD shows a rising trend^7^_._ Cardiometabolic disorders, dyslipidemia in particular, are often associated with pediatric NAFLD^8^. Approximately one-third of the Americans have NAFLD with consequent heavy economic and societal burdens^9^. Treatments for NAFLD are found to be not optimal. Some of the drugs used have side effects^10^. Hence, it would be highly desirable to devise a management plan to ascertain efficacious natural products for human populations at high risk of developing fatty liver disease in order to forestall or retard the exacerbation of hepatic damage at an early stage. To combat this disease, natural products may have value^11^.

Currently, growing attention has been drawn to the exploitation of biomedicines owing to their minimal toxicity and therapeutic efficacy. Polysaccharide, a potential agent that meets the requirement, is found in many vegetables, fruits, edible fungi and other microorganisms. Increasing studies furnish supporting evidence demonstrating that polysaccharides display a diversity of biological activities, encompassing antioxidant^12^, anti-tumor^13^, antidiabetic^14,15^, renoprotective^16,17^, immunomodulatory^18^ and hepatoprotective^19,20^ activities. Polysaccharides isolated from mushrooms have been extensively investigated in the medicinal arena owing to the ready availability of fermentation technology^21–23^.

Mushrooms exert a myriad of health promoting actions. Thus mushrooms have captured the attention of many researchers. Two important activities are hepatoprotective and antihyperlipidemic activities^24–26^. Polysaccharides from mushrooms exhibit diverse activities: those with hepatoprotective activities have been isolated from *Agaricus bisporus*, *Coprinus comatus*, *Hypsizigus marmoreus*, *Oudemansiella radicata*, *Pholiota dinghuensis*, *Pleurotus eryngii*, *Pholiota nameko*, *Pleurotus djamor*, and *Russula vinosa*^27–37^. Polysaccharides with antihyperlipidemic activity have been isolated from *Pholiota nameko* and *Termitomyces albuminosus*^38,39^.

*Auricularia polytricha*, alternatively referred to as wood ear or Jew’s ear, belonging to Auriculariaceae family, is a culinary-medicinal fungus which manifests a multitude of activities. Modern research indicates that *A*. *polytricha* is a kind of healthy mushroom with a high carbohydrate content (about 630 g/kg in dried fruiting bodies)^40^. The polysaccharides from the fruiting bodies have multiple pharmacological functions with potential for clinical application and are devoid of toxicity and significant side effects. After oral administration a soluble polysaccharide from *A*. *polytricha* exhibited antihypercholesterolemic activity in rats^41^. An ethanolic extract of mycelial culture exhibited antioxidant and tyrosinase inhibitory activities^42^. An aqueous extract of fruiting bodies demonstrated hepatoprotective effect against paracetamol in rats^43^. An aqueous extract was devoid of cytotoxicity toward normal kidney NRK-52E cells^44^ but displayed antiproliferative effect on COLO-205 colon cancer cells^45^. Polysaccharides with anticancer activity in S180 sarcoma bearing mice^46^, antimutagenic activity against the alkylating agent cyclophosphamide^47^, and antiproliferative, cell cycle arresting and apoptotic activities toward A549 human lung cancer cells^48^ have been isolated from *A*. *polytricha*. A hot water extract manifested hypoglycemic effects^49^. Hot water extracts inhibited platelet aggregation with a mechanism independent of cyclic AMP^50,51^. Thermostable constituents suppressed activity of beta secretase which liberates toxic β-amyloid peptide in the brain and protects against neurodegenerative diseases such as Alzheimer’s disease^52^. Polysaccharides with different activities have been reported from *A. polytricha*^41,46–48^. Three anticancer polysaccharides AAPS-1, AAPS-2, and AAPS-3 with molecular weights of 162, 259, and 483 kDa^46^ and an antimutagenic 930 kDa salt-soluble polysaccharide^47^ from *A*. *polytricha* have been reported.

Our previous studies indicated that polysaccharide prepared from *A*. *polytricha* effectively lowered the levels of total cholesterol, LDL-cholesterol and triglycerides^41^ in the blood circulation, indicating that the polysaccharide could regulate the metabolism of lipids, and is exploitable for alleviation of FLD and liver injury. Nevertheless, to date, no detailed studies have been carried out on the characterization, hepatoprotective effect and mechanism of polysaccharides or polysaccharide-peptides from *A*. *polytricha*. Thus we commenced this investigation on purification and characterization of bioactive polysaccharide-peptides, exploring their hepatoprotective effect and mechanism with the intent to ascertain novel bioactive constituents utilizable in the food and pharmaceutical industries.

## Results

### Extraction and purification of APPI and APPII

The crude polysaccharide-peptide from *A*. *polytricha* fruiting bodies was firstly resolved on DEAE-cellulose. According to the charge difference, two peaks, D1 and D2, eluted with 0 and 0.2 mol/L NaCl solution respectively, were detected by using the phenol–sulfuric acid method (see Supplementary Fig. S1a). The two fractions were then subjected to concentration, dialysis and gel-filtration chromatography on Superdex-200. As a result, both D1 and D2 generated only a single peak, named *A*. *polytricha* polysaccharide-peptide I (APPI) and *A*. *polytricha* polysaccharide-peptide II (APPII), respectively (refer to Supplementary Fig. S1b,c). The yields of APPI and APPII were approximately 16.31% and 49.46% (w/w) based on the weights of dried crude polysaccharide-peptide. High performance gel permeation chromatography (HPGPC) analysis disclosed that the weight-average (Mw), number-average (Mn), and z average (Mz) molecular weights and polydispersity ratio (Mw/Mn) of APPI were 9.213 × 10^5^, 5.568 × 10^5^, 1.057 × 10^6^ and 1.655, while those of APPII were 6.340 × 10^5^, 7.693 × 10^4^, 9.547 × 10^5^ and 8.241, respectively.

### Characterization of APPI and APPII

APPI and APPII exhibited the same monosaccharide moieties but different molar ratios of monosaccharides and molecular weights, similar FTIR spectra. APPI and APPII are composed of the same monosaccharide moieties, but at different ratios. Monosaccharide composition analysis revealed mannose and xylose are the major sugars, with glucose and small amounts of arabinose and galactose (see Supplementary Fig. S2a,b). APPI was composed of Ara, Gal, Glc, Xyl and Man, in a molar ratio of 1:4.4:15.4:38.3:46.2, while the corresponding molar ratio for APPII was 1:71.5:99.2:10:5.1.

**Figure 1:**
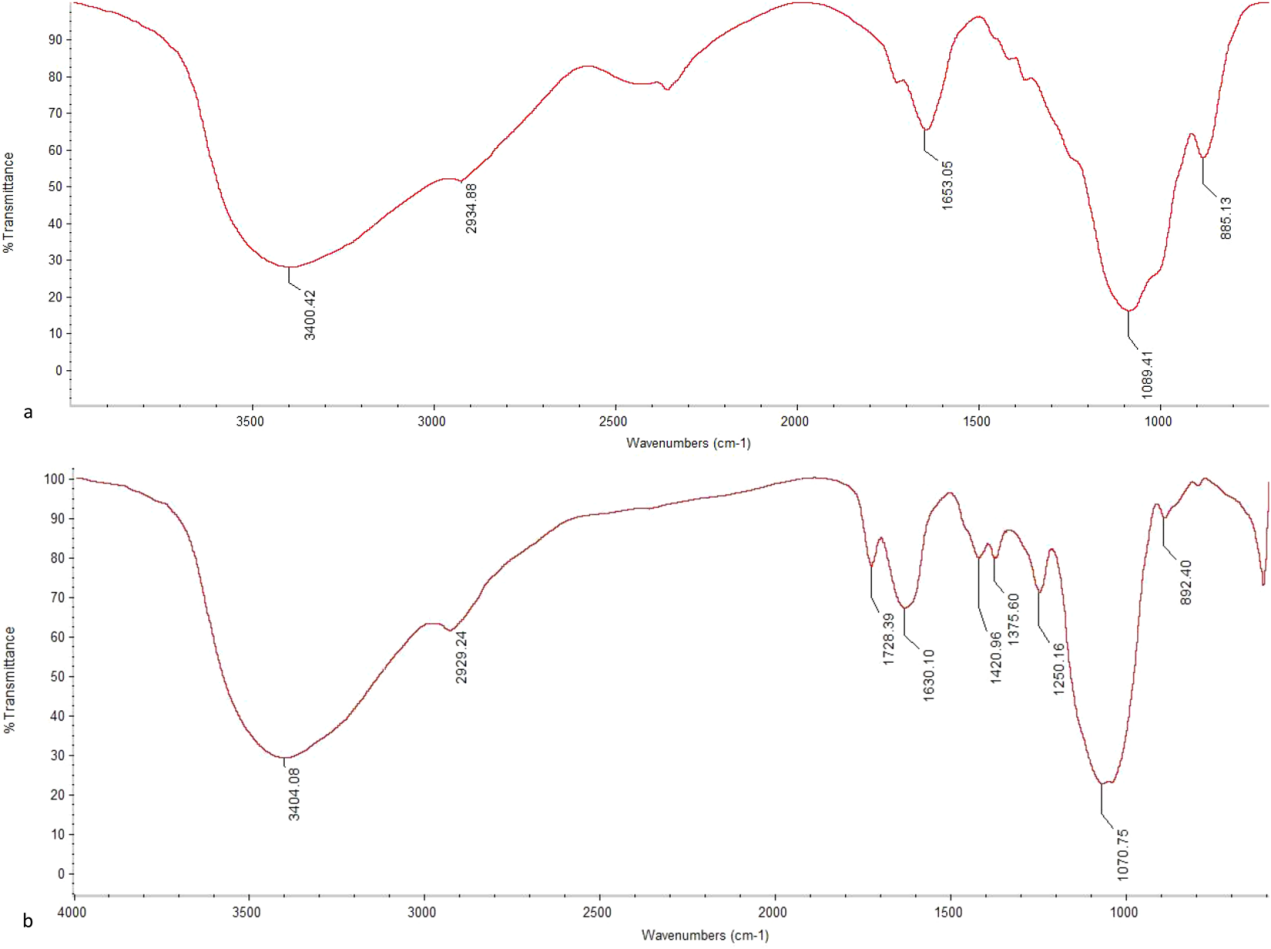
FT–IR spectrum of *A*. *polytricha* polysaccharide-peptides. (**a**) APPI, (**b**) APPII.

Fourier transform infrared spectroscopy (FTIR) is an important method to predict the structures of natural macromolecules such as polysaccharide-peptides. To more precisely characterize polysaccharide-peptide fractions, the characteristic absorption of polysaccharides was performed in the region 4000–600 cm^−1^ by FTIR spectrum. As shown in the FTIR spectrum of APPI (Fig. 1a), the intense broad peak at 3400.42 cm^−1^ was typical of hydroxyl groups with stretching vibration^53^, and the peak at 2934.88 cm^−1^ was attributed to C-H stretching vibration^54^. The peak in this region is typical of sugar. The peak at 1653.05 cm^−1^ suggested the existence of C=O bands^55^. The absorptions between 1000 and 1200 cm^−1^ were typical absorption peaks of the pyranose ring and hence disclosed the existence of C-O-H side groups and C-O-C glycosidic band vibrations^56^, and the absorption around 880 cm^−1^ to 900 cm^−1^ was attributed to the presence of α-type glycosidic linkages^57^. The FT–IR spectrum of APPII is shown in Fig. 1b, which was similar to those of APPI (Fig. 1a), but the broad absorption bands of APPII with strong intensities around 1420.96 cm^−1^ probably reflected deforming vibrations of the C-H bond. Absorption peaks were detected at 1375.60 cm^−1^and 1250.16 cm^−1^, which revealed that the polysaccharide-peptide APPII possessed a carboxyl group and a sulfate radical^58^.

The amino acid sequences of APPI and APPII at the N-terminal were DLYEVVEGEI, and VPSSMVVVVG, respectively. Results from internal amino acid sequence analysis showed that two peptide sequences of APPI, namely VQNVGNGVLLGFHGR and HQTSGDQVTSSTQHSFR, were strikingly similar to mannose-binding lectin from *Cordyceps militaris*. The peptide sequence GTPSSYIDNLTFPK of APPII manifested considerable homology with immunomodulatory protein from *Flammulina velutipes*. Another peptide sequence of APPII, ELATGQNGFGYAGSSFHR, demonstrated pronounced resemblance to peptidyl-prolyl cis-trans isomerase from *Lentinula edodes*.

### Protective effects of APPI and APPII *in vitro*

HepG2 cells of the injury model were exposed to different concentrations of FFA and ethanol, and cell viability was evaluated with the 3-(4,5-dimethylthiazol-2-yl)-2,5-diphenyltetrazolium bromide (MTT) assay. It was found that cells could retain around 60% viability when 0.5 mmol/L FFA and 1.8% (v/v) ethanol were used to induce hepatic injury. Triglyceride (TG) deposits in the cells were revealed by the Oil-red O staining method.

**Figure 2:**
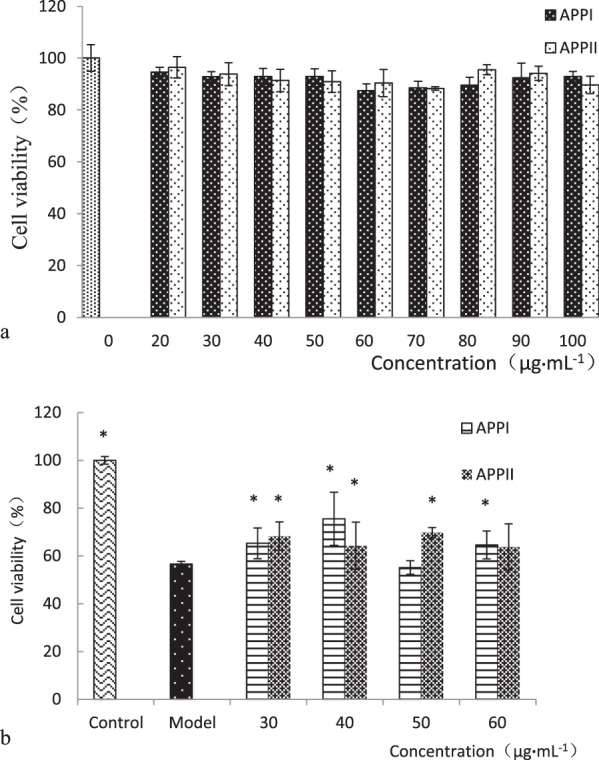
Effects of APPI and APPII *in vitro*, (**a**) Cytotoxicity, (**b**) Hepatoprotective effect. Data are shown as means ± SEM (n = 3). *P < 0.05 versus the model group as revealed by ANOVA.

The protective effects of APPI and APPII against cell injury induced by pathogenetic factors was assessed by various assays comprising the MTT test, together with assays of intracellular TG, total superoxide dismutase (SOD), and extracellular aspartate transaminase (AST) and alanine aminotransferase (ALT) activities. It was shown that APPI and APPII were not cytotoxic to HepG2 cells in the concentration range of 0–100 μg/mL (Fig. 2a). The cells were exposed to increasing concentrations of APPI or APPII (30–60 μg/mL) for 24 h after the addition of 0.5 mmol/L FFA and 1.8% (v/v) ethanol. As shown in Fig. 2b, FFA and ethanol treatment brought about significant toxicity to HepG2 cells, whereas treatment with APPI or APPII increased cell viability with the highest ratio of 33.47% at 40 μg/mL and 23.23% at 50 μg/mL, respectively. The results showed that intracellular TG in APPI and APPII groups was attenuated relative to that in the model group (P < 0.05), as shown in Table 1, which was also verified through morphological observation aided by the Oil-red O stain (see Supplementary Fig. S3). APPI and APPII elevated the activity of total SOD in HepG2 cells considerably, by 72.72% and 58.43%, relative to the model group (Table 1). The most conspicuous effect of hepatic injury is the liberation of intracellular enzymes, like aminotransferases, into the circulation, which ensues in an escalation of the activities of these enzymes^6^. Thus, activities of extracellular aminotransferases can reflect the status of liver cells, with higher values reflecting liver damage. It was shown that the activities of extracellular alanine and aspartate aminotransferases (ALT and AST) of the model group were elevated relative to those of the control group, implying that pathogenetic factors of FFA and ethanol inflicted damage on the cells. However, APPI and APPII mitigated this damage, and the elevated activities of alanine and aspartate aminotransferases consequently declined (P < 0.05), as shown in Table 1.

**Table 1:** Protective effect of APPI and APPII reflected in cellular indexes.

Groups	TG (µmol/mg protein)	T-SOD (U/mg protein)	ALT (U/L)	AST (U/L)
Control	261.48 ± 25.19*	24.22 ± 0.84*	11.34 ± 0.48*	9.43 ± 0.92*
Model	745.51 ± 59.22	11.63 ± 1.24	23.19 ± 0.76	21.29 ± 0.58
APPI	604.04 ± 58.07*	20.08 ± 1.23*	15.24 ± 1.11*	13.79 ± 0.43*
APPII	472.84 ± 49.46*	18.41 ± 0.98*	18.92 ± 0.29*	15.34 ± 0.17*

Data are shown as means ± SEM (n = 3).

*P < 0.05 versus the model group as revealed by ANOVA.

### Mechanisms of APPI and APPII

Adiponectin is an adipocyte-derived cytokine. Adiponectin and its receptor 2 abundant in human hepatocytes, constitute a complex, and play a central role in the pathogenesis of liver disorders. Adiponectin, which has an anti-steatotic effect on liver cells, acts mainly through the AMPK and PPARα pathway. It promotes oxidation of FFA and suppresses gluconeogenesis, entry of FFA and *de novo* lipid synthesis^7,8^. When drugs stimulate the adipocytes, adiponectin is secreted and conjugated with Adipor 2 receptors on the surface of the hepatocytes. The complex activates *AMPK* gene expression, induces genes including *CPTl* and *ACOX1* in FFA oxidation pathway upregulation. Meanwhile, *AMPK* induces activation of transcription factor *PPARα* and accelerates FFA metabolism.

**Figure 3:**
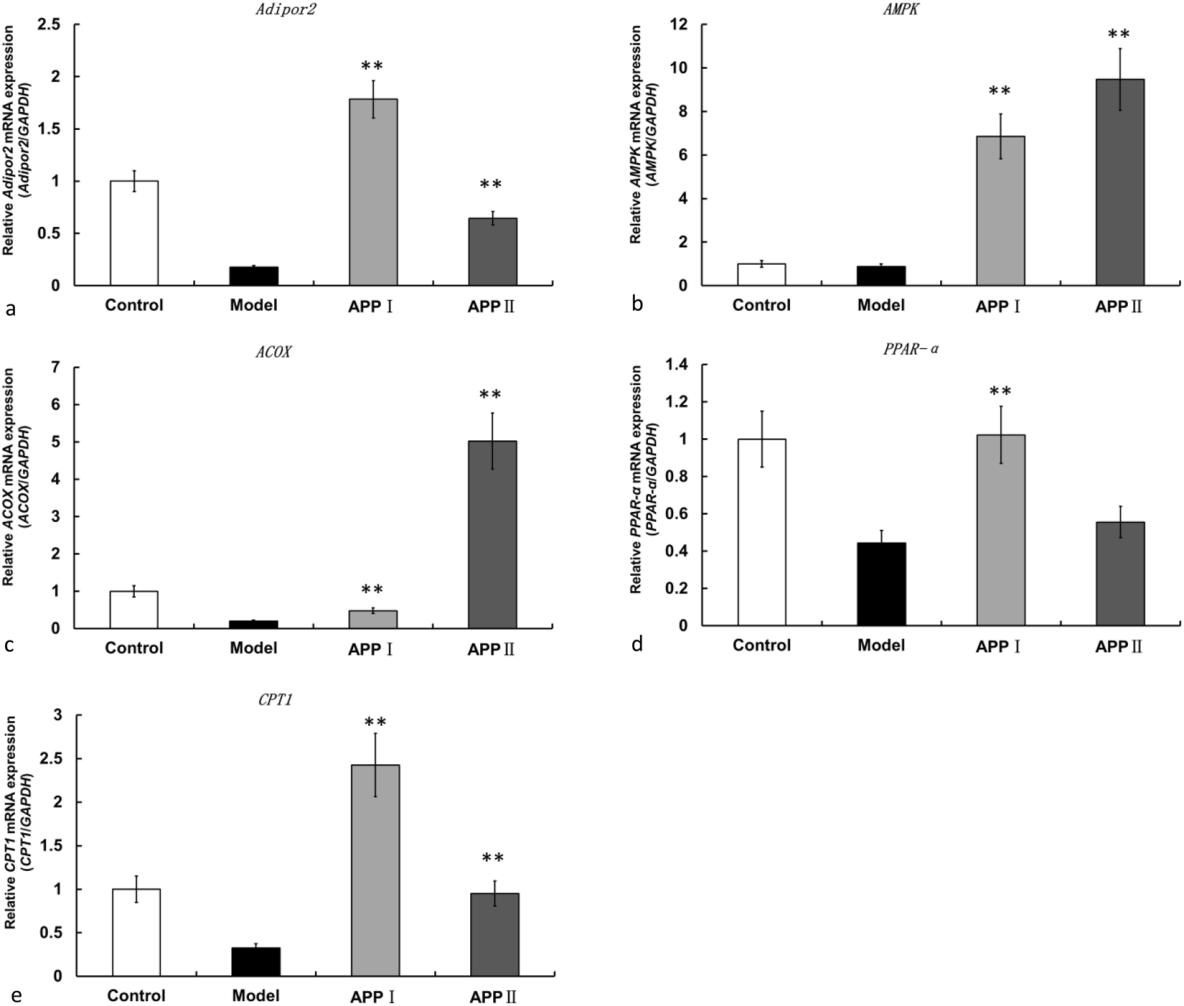
q-PCR analysis of APPI and APPII on expression of genes in the adiponectin pathway. Data are shown as means ± SEM (n = 3). **P < 0.01 versus the model group as revealed by ANOVA.

In order to validate the mechanism of APPI and APPII, five key genes have been selected for qRT-PCR analysis (Fig. 3). The results showed that the qRT-PCR data of these genes were consistent with results of the bioassays. For example, *Adipor 2* gene was significantly down-regulated in the model group, as well as *AMPK*, *CPTl*, *PPARα* and *ACOX1*, which deactivate FFA oxidation process and accelerate FFA accumulation in hepatocytes or hepatic tissue. It was disclosed that the TG concentration of the model group rose. It was found that *Adipor 2* gene was up-regulated in APPI and APPII groups, the same took place regarding *AMPK*, *CPTl*, *PPARα* and *ACOX1*. The results indicated that APPI and APPII could stimulate adipocytes to secrete adiponectin and activate the FFA metabolic pathway, reducing lipid accumulation.

### Hepatoprotective effect of APPI and APPII *in vivo*

#### Protective effects of APPI and APPII on hepatic damage

FLD is the leading cause of chronic liver disease worldwide. Unhealthy high fat diets and sedentary habits result in the high prevalence of FLD. The rats were randomized to receive either standard laboratory diet (SLD) or high cholesterol and high fat diet (HFD) for 8 weeks. The biochemical analysis showed that HFD induced a rise in the levels of TG, total cholesterol (TC) and low-density lipoprotein cholesterol (LDL-C), and the activities of ALT and AST in serum (see Supplementary Table S1). The HFD rats gained weight faster than those with SLD, at the ratio of 105.43%, and the liver weights in the model groups were heavier than that in control group. AST and ALT activities in the serum have been employed as biochemical markers for hepatic damage. The heightened AST and ALT expression levels signified the enhanced permeability and injury of hepatocytes.

In the present study, APPI and APPII ameliorated the biochemical indexes. Significant decreases (P < 0.05) in ALT and AST activities were observed in APPI and APPII-treated groups similar to the simvastatin- treated group (Table 2). It indicated that APPI and APPII could protect hepatocytes from injury and maintain their integrity. The concentrations of TC, TG, LDL-C, and high density lipoprotein cholesterol (HDL-C) in the serum are vital parameters in the analysis of lipid metabolism. Abnormal serum lipid indicated the risks of FLD. The TC, TG and LDL-C concentrations of serum in APPI or APPII-treated groups were reduced relative to those in the model group (P < 0.05). It was indicated that APPI and APPII might be useful for preventing FLD through improving lipid metabolism.

**Table 2:** Hepatoprotective effects of APPI and APPII on serum biochemical markers.

	TG (mmol/L)	TC (mmol/L)	HDL-C (mmol/L)	LDL-C (mmol/L)	AST (U/L)	ALT (U/L)
Control group(SLD)	0.92 ± 0.23*	2.36 ± 0.28*	1.52 ± 0.19*	0.44 ± 0.09*	133.00 ± 16.54*	136.60 ± 24.31*
Model group(HFD)	1.96 ± 0.21	3.98 ± 0.44	1.01 ± 0.11	2.78 ± 0.43	215.40 ± 15.84	233.00 ± 19.72
APPI + HFD group (50 mg/kg/d)	0.44 ± 0.08*	2.48 ± 0.08*	1.46 ± 0.14*	0.66 ± 0.13*	148.50 ± 20.41*	160.00 ± 14.83*
APPI + HFD group (100 mg/kg/d)	0.39 ± 0.09*	2.20 ± 0.3*	1.59 ± 0.21*	0.60 ± 0.08*	151.00 ± 13.18*	172.33 ± 27.26*
APPII + HFD group (50 mg/kg/d)	0.33 ± 0.03*	2.15 ± 0.24*	1.37 ± 0.33	0.57 ± 0.06*	211.33 ± 15.75	215.67 ± 12.93
APPII + HFD group (100 mg/kg/d)	0.24 ± 0.03*	1.73 ± 0.33*	1.11 ± 0.22	0.50 ± 0.10*	148.33 ± 7.95*	126.83 ± 3.99*
Simvastatin (2 mg/kg/d)	0.64 ± 0.08*	2.02 ± 0.25*	1.19 ± 0.17	0.73 ± 0.08*	109.33 ± 26.63*	101.00 ± 21.57*
HFD-SLD group	0.91 ± 0.12*	2.16 ± 0.11*	1.34 ± 0.08*	0.67 ± 0.03*	115.20 ± 14.01*	121.80 ± 30.24*

Data are shown as means ± SEM (n = 6). *P < 0.05 versus the model group as revealed by ANOVA.SLD = standard laboratory diet. HFD = high fat diet.

**Figure 4:**
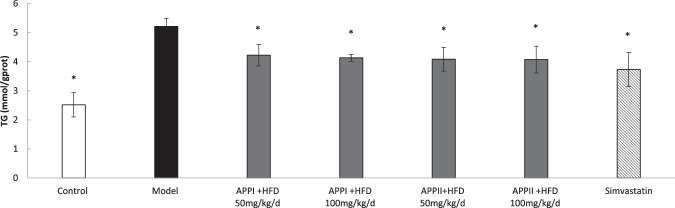
Hepatic TG content in rats. Data are shown as means ± SEM (n = 6). *P < 0.05 versus the model group as revealed by ANOVA. Control rats were fed standard laboratory diet (SLD)while the rest were fed a high fat diet (HFD).

HFD is associated with fat accumulation in liver. As shown in Fig. 4, hepatic TG level was elevated significantly by 2.07 fold versus the control group. However, treatment of HFD with APPI and APPII, just like treatment with simvastatin, significantly (P < 0.05) lowered the TG level in liver tissue compared to the model group.

### Histopathological observations

**Figure 5:**
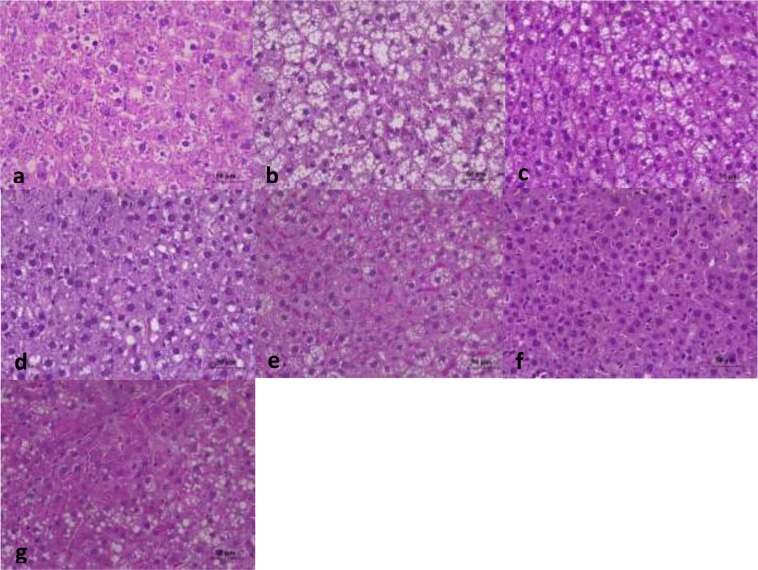
Histopathological alterations in rat livers, stained with H&E (400×). (**a**) Control group, (**b**) Model group, (**c**) APPI + HFD group (50 mg/kg/d), (**d**) APPI + HFD group (100 mg/kg/d), (**e**) APPII + HFD group (50 mg/kg/d), (**f**) APPII + HFD group (100 mg/kg/d), (**g**) Simvastatin group (2 mg/kg/d). Control rats were fed standard laboratory diet (SLD) while the rest were fed a high fat diet (HFD).

Histopathological examination was used to evaluate the hepatoprotective effects of APPI and APPII on HFD-elicited acute liver lesion. Figure 5b revealed that the nuclei of hepatocytes in the model group were irregular, lysis diffluence and even disappearance. The cytoplasm exhibited a ballooning degeneration with fatty droplets. It was also observed that hepatic sinus appeared eclasis and gore, some of Kupffer cells proliferated. It suggested that the tissues in the model group turned to hepatocyte injury and the FLD model was successfully established. However, the hepatocytes showed a regular arrangement, the cytoplasmic ballooning degeneration was mitigated in APPI and APPII with HFD groups (Fig. 5c–f). In the APPI and APPII treated groups, lipid and hepatocyte injury were reduced relative to the model group. Hepatocyte volume was reduced, liver lobules were distinctly delineated, and there were fewer fat droplets. These histological alterations were in agreement with the serum and liver lipid profiles shown in Fig. 4 and Table 2. Therefore, APPI and APPII minimized lipid deposition in the liver cells of FLD rats.

## Discussion

In our earlier study a soluble polysaccharide was prepared from *A*. *polytricha* fruiting bodies with an extraction rate approximating 20% by using the following conditions: extraction with 28 mL water/g mushroom at 95 °C for 4 h. The polysaccharide preparation exerted an antihypercholesterolemic action in diet-induced hypercholesterolemic rats and depressed the plasma cholesterol level by about one-third at the doses of 4.5 and 9 mg/kg/d. In the present investigation the polysaccharide was fractionated into two polysaccharide-peptides APPI and APPII have similar monosaccharide moieties and FITR spectra but differ in molar ratio of the monosaccharides and molecular weight. APPII also differs from APPI in exhibiting absorption around 1420.96 cm^−1^ probably indicating deforming vibrations of the C-H bond and absorption at 1375.60 cm^−1^ and 1250.16 cm^−1^ signifying the presence of a carboxyl group and a sulfate radical. Although APPI and APPII possess distinct N-terminal and internal partial amino acid sequences, both of them resemble other mushrooms in these sequences. In APPI, two of the internal partial amino acid sequences closely resemble those of *Cordyceps militaris* mannose-binding lectin. In APPII, two of the internal partial amino acid sequences manifest pronounced similarity to those of *Flammulina velutipes* immunomodulatory protein and *Lentinula edodes* peptidyl-prolyl cis-trans isomerase, respectively.

*Auricularia auricular* polysaccharides simulated hydrolysates, derived from *A*. *auricular* which is a species very closely similar to *A*. *polytricha*, were composed of arabinose, galactose, glucosamine, glucose, xylose and mannose with the molar ratio of 1.91: 0.67:0.23:1.00: 0.52: 2.89^59^. *A*. *auricular* polysaccharide was composed of arabinose, galactose, glucosamine, glucose, rhamnose and mannose with the molar ratio of 0.93:0.91:4.32:1:37.53^60^. APPI isolated in the present study was composed of arabinose, galactose, glucose, xylose and mannose in a molar ratio of 1:4.4:15.4:38.3:46.2, while the corresponding molar ratio for APPII was 1:71.5:99.2: 10: 5.1. Glucosamine was lacking in APPI and APPII. The data showed that they were all different in the composition of the sugar moiety.

The FT-IR spectrum of APPI exhibited a broad peak at 3400.42 cm^−1^ characteristic of hydroxyl groups with stretching vibration, and a peak at 2934.88 cm^−1^ due to C-H stretching vibration. The absorption peak at 1250.16 cm^−1^ revealed that the polysaccharide-peptide APPII possessed a sulfate radical. In the study of Zhang *et al*.^61^, the FT-IR spectrum of *A*. *auricular* polysaccharide also displayed a band in the region of 3411.1 and 3410.5 cm^−1^ corresponding to the hydroxyl stretching vibration of the polysaccharide and at 2923.3 and 2921.3 cm^−1^ corresponding to a weak C–H stretching vibration at 1258 cm^−1^ due to an asymmetrical S=O stretching vibration. Although there were similarities the spectra of the aforementioned polysaccharides were not identical.

With regard to its effect on lipid metabolism, an aqueous extract of *A*. *polytricha*, which was composed of phenolics, tannins and polysaccharides, was found to reduce lipid storage, inflammation, proinflammatory cytokines and oxidative stress in rats provided with a high-fat diet containing 10% lard. Upon completion of the treatment which lasted for 12 weeks. It was observed that changes brought about by the high-fat diet including the hepatosomatic index, serum activities of aminotransferases, triglyceride, serum levels of free fatty acid, total cholesterol, high density lipoprotein cholesterol, low density lipoprotein cholesterol, very low density lipoprotein cholesterol, antioxidant vitamins C and E, malondialdehyde, and the proinflammatory cytokines interleukin-6 and tumor necrosis factor-alpha, were at least partially reversed by treatment with the *A*. *polytricha* polysaccharide-peptides^62^. Alterations in hepatic triglyceride, cholesterol, malondialdehyde, antioxidant vitamins E and C, and antioxidant enzymes were also attenuated^62^. Intake of a *Ganoderma lucidum* preparation enriched in polysaccharide-peptides and triterpenoids by human subjects restored the hepatic condition to normal from the initially mild fatty liver as evidenced by abdominal ultrasonography and reduction in plasma activities of aminotransferases^63^. However, it is known that phenolics and tannins also displayed similar activities^64^. Thus, the aforementioned actions of the aqueous extract of *A*. *polytricha*^62^ were probably attributed to each of its constituents: phenolics, tannins and polysaccharides, and the action of the *Ganoderma lucidum* preparation^63^ was partly due to triterpenoids^65,66^.

In the present investigation both APPI and APPII exhibited a lipid lowering effect *in vitro* as well as *in vivo*. In the *in vitro* system employing ethanol and a mixture of palmitic and oleic acids to induce hepatotoxicity, the resulting elevated level of intracellular triglyceride and activity of extracellular aspartate and alanine aminotransferase were suppressed by APPI and APPII, and the decreased cellular superoxide dismutase activity was upregulated by the two polysaccharide-peptides. APPI and APPII increased the viability of the hepatocytes. It appeared that APPII was more potent in inhibiting triglyceride accumulation in the hepatocytes. However, this was not true regarding the effects on hepatocyte viability, superoxide dismutase activity, and aminotransferases released into the culture medium. APPI had a more potent effect on *Adipor 2*, *CPTl*, and *PPARα* whereas APPI had a higher activity on *AMPK* and *ACOX1*.

In the *in vivo* system, the high fat diet brought about a rise in the circulatory titers of triglyceride, total cholesterol, low density cholesterol, aspartate aminotransferase and alanine aminotransferase but a decline in plasma high density cholesterol. APPII was more potent than APPI in its antihypercholesterolemic and antihypertriglyceridemic and LDL lowering activities. APPI was more potent than APPII in raising plasma HDL cholesterol activity. APPI and APPII were similar in their activity in preventing hepatic triglyceride accumulation and secretion of aspartate aminotransferase and alanine aminotransferase into the blood stream. The hepatocytes after treatment with APPI and APPII were similar in appearance under the microscope.

Polysaccharides from *A*. *auricula* very closely similar to *A*. *polytricha* also reduced the serum concentrations of total cholesterol and low-density lipoprotein cholesterol in mice receiving a diet enriched in cholesterol and improved lipoprotein lipase activity and total antioxidant capacity^67^. A water-soluble crude polysaccharide from *A*. *auricular* mycelia grown under solid-state fermentation lowered serum concentrations of triglyceride, total cholesterol, and low-density lipoprotein cholesterol in a high fat diet induced hyperlipidemic mice^60^. *A*. *auricular* polysaccharides simulated hydrolysates from the dried fruiting bodies induced a decline in the serum concentrations of triglyceride and low-density lipoprotein cholesterol without affecting high-density lipoprotein cholesterol and total cholesterol in streptozotocin -induced diabetic rats^59^. The alcohol extract of *A*. *auricula*-judae provided to mice on a high fat diet reduced plasma lipids and hepatic enzymes and downregulated expression of adipogenic/lipogenic genes (PPARγ, C/EBPα, FAS) in 3T3-L1 cells^68^. An alcohol extract of *A*. *auricula* enriched in polyphenolics lowered serum total cholesterol and upregulated high-density lipoprotein cholesterol level and fecal bile acid excretion. However, it was devoid of any action on serum low-density lipoprotein cholesterol and triglycerides and fecal neutral cholesterol excretion^59^. The effects of *Auricularia cornea* polysaccharides and enzymatic-extractable polysaccharides displayed antioxidant and reactive oxygen species scavenging activities *in vitro* and exerted a protective action on alcohol-induced liver pathology in mice. The polysaccharides produced their hepatoprotective effects by suppressing lipid peroxidation, facilitating alcohol metabolism, and downregulatiing the expression of inflammatory mediators and preventing the alcohol-induced histopathological alterations^69^.

Adiponectin is anti-inflammatory and insulin-sensitizing adipokine. Adiponectin deficiency linked with an inflammatory state is present in obesity, severe chronic hepatitis C-related steatosis, non-alcoholic fatty liver diseases such as hepatic steatosis and non-alcoholic steatohepatitis, and cancer^70–74^. Adiponectin reduces insulin resistance, prevents excess hepatic lipid accumulation, inhibits inflammation and fibrosis, and exerts a hepatoprotective action^75–77^. It also protects against cerebrovascular, cardiovascular, and chronic renal diseases^7,71,72,76–80^. It is a target in the therapy of obesity, cardiovascular and inflammatory ailments, nonalcoholic steatohepatitis, diabetes and neurodegenerative diseases^7,76,78–80^. Efforts are made to upregulate adiponectin by therapeutic medications and/or changes in lifestyle^77^.

Mushroom extracts exhibit a stimulatory effect on adiponectin production. An elevation of the circulatory adipokine level, decline in levels of markers associated with hepatic damage, and activities of key enzymes catalyzing fatty acid biosynthesis, together with attenuation of liver enlargement and triglyceride accumulation, were observed in *db/db* mice after consumption of ethanol-soluble extract of *Panellus serotinus*^81,82^. Supplementation with *Agaricus blazei* reduces insulin resistance and improves circulatory adiponectin level in patients with type 2 diabetes Murill^83^. β-glucan-rich polysaccharides derived from *Pleurotus sajor-caju* upregulated adiponectin expression^84^. The ethyl acetate fraction of *Hericium erinaceus* downregulated lipopolysaccharide -elicited decline of adiponectin mRNA in 3T3-L1 fat cells in coculture with RAW264 macrophages^85^. An aqueous extract of *Antrodia cinnamomea* reversed the action of a high-fat diet on body weight gain, inflammatory cytokines and adiponectin production in mice^86^. Adiponectin promotes fatty acid oxidation in skeletal muscle^79^. The purified polysaccharide- peptides acquired from *A*. *polytricha* act via genes affect fatty acid oxidation. Peroxisome proliferator-activated receptors (PPARs) are metabolic regulators of lipid and lipoprotein levels which are divided into α, β/δ and γ subtypes. The PPAR-α agonists suppress triglyceride levels. PPAR-γ agonists demonstrate potent hypoglycemic but weaker triglyceride lowering activity. PPAR-α/δ agonists display antihyperglycemic, and triglyceride lowering activities. They are promising for the therapy of atherogenic dyslipidemias and NAFLD^87^. PPARs are paramount to energy homeostasis of the entire body and as fatty acids sensors in several human lipid metabolic diseases^88^. Extracts, polysaccharides and other compounds from mushrooms inhibit fat production by acting via PPAR γ and/or PPAR α. This study is the first to reveal the action of mushroom polysaccharide peptides through PPAR α.

The present report on the hepatoprotective and fatty liver alleviating activities of purified polysaccharide-peptides acquired from *A*. *polytricha* and elucidation of the mechanism involved represents the first of its kind on purified polysaccharide-peptides. The *A*. *polytricha* polysaccharide peptides are of considerable interest and promising for development into therapeutic agents in view of the array of health-enhancing activities of adiponectin.

## Methods

### Materials and reagents

*A*. *polytricha* fruiting bodies were collected in Beijing, China, and were ground to produce a fine powder. Monosaccharides (D-mannose, D-glucose, D-glucuronic acid, L-rhamnose, D-xylose, D-fructose, D-galacturonic acid, D-galactose and D-arabinose), DEAE-cellulose, MTT, oleic acid and palmitate were products of Sigma-Aldrich (USA). Superdex-200 column was obtained from General Electric Company (GE, USA), and HepG2 cell line was purchased from ATCC (American Type Tissue Culture Collection). Dulbecco’s modified Eagle’s minimum essential medium (DMEM), fetal bovine serum (FBS), phosphate buffered saline (PBS), trypsin solution, penicillin and streptomycin were products of Invitrogen (USA). The assay kits for protein content, TG, ALT, AST, and SOD were purchased from Nanjing Jiancheng Bioengineering Institute (Nanjing, Jiangsu Province, China). The other chemicals and solvents employed were of analytical reagent grade and obtained from Sinopharm Chemical Reagent Co., Ltd. (Shanghai, China).

### Extraction and isolation of polysaccharide-peptides

Crude polysaccharide-peptides were extracted from *A*. *polytricha* following published procedures^41^. Briefly, the dry powder was extracted thrice, for 4 h each time, by employing 40 volumes of hot water (90 °C). The extracts were combined and concentrated in a rotary evaporator. The concentrated solution was deproteinated using Sevag reagent (chloroform and n-butanol, 4:1 vol:vol)^89^. Ethanol (100%) was added to the deproteinated solution which was then allowed to stand at 20 °C overnight, and the precipitated polysaccharide-peptide (APP) was obtained by centrifugation. Subsequently, a solution of the APP in distilled water was fractionated on a DEAE-cellulose column (1 cm × 30 cm) equilibrated with distilled water. The column was eluted sequentially with 0, 0.2 and 1 mol/L NaCl solution successively at a flow rate of 2.0 mL/min. The unadsorbed peak D1 and adsorbed peak D2, with the carbohydrate content measured with the phenol-sulfuric acid method, were enriched in polysaccharide. The D1 and D2 fractions were collected, concentrated, and further fractionated on an FPLC-Superdex 200 10/300 column in 0.2 mol/L NH_4_HCO_3_ (pH 8.5) buffer using an AKTA Purifier (GE Healthcare), respectively to obtain bioactive polysaccharide-peptides APPI and APPII.

### Characterization of bioactive polysaccharide-peptides

#### Analysis of monosaccharide composition

Monosaccharide compositions of APPI and APPII were determined with a gas chromatography mass spectrometer (GC-MS). The sample was hydrolyzed following the method of Yu *et al*.^90^.

#### FT–IR analyses

The IR spectra of APPI and APPII were obtained by using a Fourier transform infrared spectrophotometer (Nicolet iS5 FTIR Spectrometer, USA) within the wave number range of 4000 to 400 cm^−1^. The dried sample was ground with spectroscopic grade potassium bromide (KBr) powder and then pressed into 1- mm pellets.

#### Determination of molecular weight

The purity and molecular weights of APPI and APPII were assessed with HPGPC technique using HPLC on a TSK GMPWXL column. The samples were injected and eluted at 0.6 mL/min with 0.1 mol/L NaNO_3_ and 0.05%NaN_3_ as the mobile phase.

#### N-terminal and inner amino acid sequence analysis

The polysaccharide-peptides band excised from the SDS-PAGE gel was transferred to a polyvinylidenedifluoride (PVDF) membrane by Western blotting and then stained with Coomassie brilliant blue R-250. Analysis of the stained band was performed with the automated Edman degradation method^91^.

The polysaccharide-band of SDS-PAGE gel was recovered and dispatched to Tsinghua University (Beijing, China) for partial amino acid sequence analysis. Sequence homology with known sequences was searched using the BLAST/NCBI database.

### Assay for the protective effects of APPI and APPII on hepatocytes

#### Preparation of model of hepatocyte injury-induced by ethanol and a mixture of palmitic and oleic acids

FFA and ethanol were considered as the pathogenetic factors which induce hepatocyte injury. FFA used was a mixture of palmitic and oleic acids, prepared according to the method described by Garcia *et al*.^92^. The hepatocyte injury model was established as follows. HepG2 (liver cancer) cells were cultured in Dulbecco’s Modified eagle medium (DMEM) medium containing 10% (v/v) fetal bovine serum (FBS), 100 IU/mL penicillin and 100 mg/L streptomycin, at 37 °C in a humidified atmosphere of 5% (v/v) CO_2_. Cells were then seeded onto 96-well plates at a concentration of 8 × 10^3^ cells/well, and allowed to incubate for 12 h before addition of FFA and ethanol. Incubation was then conducted for 24 h. Afterwards, the MTT assay was performed to measure cell viability. As a control, PBS instead of pathogenetic factors (FFA and ethanol) was added to the wells.

#### Protective effects and mechanisms of APPI and APPII on hepatocytes

To determine hepatoprotective effects, injured HepG2 cells were treated with APPI and APPII at various concentrations for 48 h. Upon termination of the incubation, viability of the cells was measured with the MTT assay. The optimal doses, obtained by using cell viability determination, were considered as effective concentrations to analyze the mechanism of APPI and APPII. HepG2 cells were seeded onto 6-well plates at a concentration of 3 × 10^4^ cells/well, for preparation of the injury models before addition of APPI and APPII at optimal doses. Following incubation for 48 h, the cells and culture medium in all treatment groups were collected to evaluate the repairing ability. Intracellular protein, cellular TG and the activities of SOD from cells were quantified using commercial assay kits, and the activities of ALT and AST in the culture medium were assayed with colorimetric assay kits, all in accordance with the manufacturers’ protocols. Histological analysis of cellular lipid was determined with the Oil-red O stain method.

#### Gene expression analysis

To analyze the therapeutic pathways of APPI and APPII, the expression levels of lipid metabolism genes in the adiponectin pathway were determined by qRT-PCR. Extraction of total RNA from the samples was conducted by using TRIzol reagent (Invitrogen, USA) and reversely transcribed with oligo (dT) using EasyScriptTM First-Strand cDNA Synthesis SuperMix (Transgen, China), in accordance with the manufacturer’s protocol. qRT-PCR was performed following the protocol of the Maxima SYBR Green/ROX qRT-PCR Master Mix (Fermentas, USA) employing an ABI 7500 (Applied Biosystems, USA). The GAPDH RNA level was used as an endogenous control for mRNAs. The qRT-PCR procedure comprised pre-denaturation at 95 °C for 5 min and 40 cycles which consisted of exposure to 95 °C for 30 seconds, and to 60 °C for 1 min. The relative expression level was computed by the 2-ÄÄCt method. Five independent experiments were performed.

### Protective effects of APPI and APPII against fat-induced fatty liver in rats

#### Experimental animals and diets

Male Wistar rats (220–280 g) obtained from the Institute of Laboratory Animal Science, Chinese Academy of Medical Sciences, were utilized in this study. All experimental protocols were approved by the University Safety Office and Animal Experimentation Ethics Committee at The Chinese University of Hong Kong and China Agricultural University. All animal experiments were carried out in accordance with the approved guidelines of the Animal Care and Use Committee of The Chinese University of Hong Kong and China Agricultural University. The normal control group was fed with SLD, while the other groups received a HFD including 2% cholesterol and 25% pig fat.

#### Animal grouping and experimental design

Rats were randomly allocated in seven groups with six rats in a group. The normal control group was fed SLD, while the other six groups received a HFD including 2% cholesterol and 25% pig fat for eight weeks. The six HFD groups were treated as follows: (i) the model group (no treatment), (ii) low-dose APPI group (50 mg/kg/d), (iii) high-dose APPI group (100 mg/kg/d), (iv) low-dose APPII group (50 mg/kg/d), (v) high-dose APPII group (100 mg/kg/d), (vi) positive group of simvastatin treatment (2 mg/kg/d). APPI, APPII and simvastatin were administered intragastrically for 4 weeks after an 8-weeks HFD model foundation. Rats in the normal control group and model control group an equal volume of normal saline instead. Free access to food and water was allowed. The rats were weighed weekly, and fresh food was provided daily.

After the final drug treatment, the animals were sacrificed by cervical dislocation, and blood was collected for biochemical analysis. The liver was removed and weighed. A small piece of the liver was fixed in 10% buffered formalin solution for histological processing, and the remainder was frozen at −80 °C till biochemical analysis.

#### Histological analysis and biochemical evaluation

The hepatic tissues were preserved in 4% paraformaldehyde at 4 °C for 24 h before embedding in paraffin for sectioning. The tissue sections were prepared and stained with hematoxylin and eosin (H&E). Hepatic histology was observed using an Olympus microscope, IX71 (Olympus, Japan).

Liver tissue was homogenized using phosphate buffer saline. Following centrifugation at 10,000 r/min for 40 min at 4 °C, the supernatant was saved for biochemical evaluation. TC and TG levels as lipid indexes, and ALT and AST activities as hepatic injury indexes were assayed using commercial kits following the manufacturer’s instructions.

#### Statistical analysis

All experiments in all of the bioassays were conducted in triplicate. Data are shown as mean ± standard deviation (SD). Statistical analysis was conducted with one-way analysis of variance (ANOVA) using SPSS software (SPSS 16.0 software package, USA). Data were assessed by using ANOVA and P ≤ 0.05 was considered to be statistically significant.

## Conclusion

The present report on the hepatoprotective and fatty liver alleviating activities of purified polysaccharide-peptides acquired from *A*. *polytricha* and elucidation of the mechanism involved represents the first of its kind on purified polysaccharide-peptides. The *A*. *polytricha* polysaccharide peptides are of considerable interest and promising for development into a therapeutic agent in view of the array of health-enhancing activities of adiponectin.

## Supplementary information

Supplementary information
