# Supplementary material for: First demonstration of protective effects of purified mushroom polysaccharide-peptides against fatty liver injury and the mechanisms involved
Source: Sci Rep. 2019 Sep 23;9:13725. doi: 10.1038/s41598-019-49925-0 (PMC6757109; doi:10.1038/s41598-019-49925-0)
Supplement: Supplementary file 1 — Supplementary information [file 41598_2019_49925_MOESM1_ESM.pdf]

First demonstration of protective effects of purified mushroom polysaccharide-peptides against fatty liver injury and the mechanisms involved

Shuang Zhao[ab*], Shuman Zhang[a*], Weiwei Zhang[c*], Yi Gao[d], Chengbo Rong[a], Hexiang Wang[e#], Yu Liu[a], Jack Ho Wong [f#],Tzibun Ng[f#]

[a] Institute of Plant and Environment Protection, Beijing Academy of Agriculture and Forestry Sciences, Beijing 100097, China

[b]Beijing Key Laboratory of Fruits and Vegetable Storage and Processing, Key Laboratory of Vegetable Postharvest Processing, Ministry of Agriculture, Beijing 100097, China

[c]Institute of Medicinal Plant Development, Chinese Academy of Medical Sciences & Peking Union Medical College, Beijing 100193, China

[d]Beijing Xicheng District Health Care Center for Mothers and Children，Beijing 100053, China

[e]State Key Laboratory for Agrobiotechnology and Department of Microbiology, China Agricultural University, Beijing 100193, China

[f]School of Biomedical Sciences, Faculty of Medicine, The Chinese University of Hong Kong, Shatin, New Territories, Hong Kong, China

Email addresses: shuangzhaow@163.com (S Zhao)

zhang_shu_man@163.com（S Zhang）

wwzhang@implad.ac.cn (W Zhang)

13439023411@126.com (Y Gao)

woshiboer@163.com （C Rong）

hxwang@cau.edu.cn (HX Wang)

ly6828@sina.com (Y Liu)

jack1993@yahoo.com (JW Wong)

b021770@mailserv.cuhk.edu.hk (TB Ng)

---

[*]These authors contributed equally to this work.

[#] Corresponding authors

Email: hxwang@cau.edu.cn (HX Wang)
Tel: +86 10 62732578
Fax: +86 10 62732578
Email: jack1993@yahoo.com (JW Wong)
Email: b021770@mailserv.cuhk.edu.hk (TB Ng)

1. *Purification of APPI and APPII*

The crude polysaccharide-peptide was firstly resolved on a DEAE-cellulose anion-exchange column. According to the charge difference, two peaks, D1 and D2, eluted with 0 and 0.2 mol/L NaCl solution respectively, were detected by using the phenol–sulfuric acid method (shown in SIFig.1a). The two fractions were then collected, concentrated, dialyzed and subjected to further purification by gel-filtration chromatography on a Superdex-200 column. As a result, both D1 and D2 generated only a single peak, named APPI and APPII, respectively (shown in SIFig.1b,c).

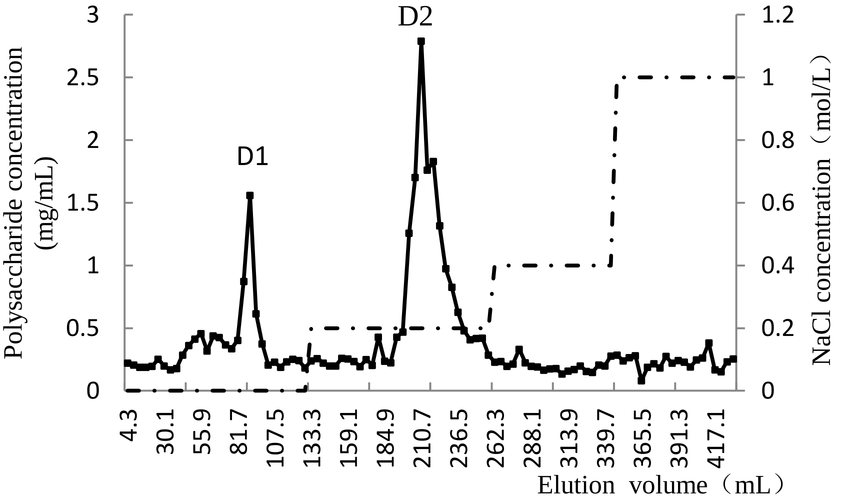

a

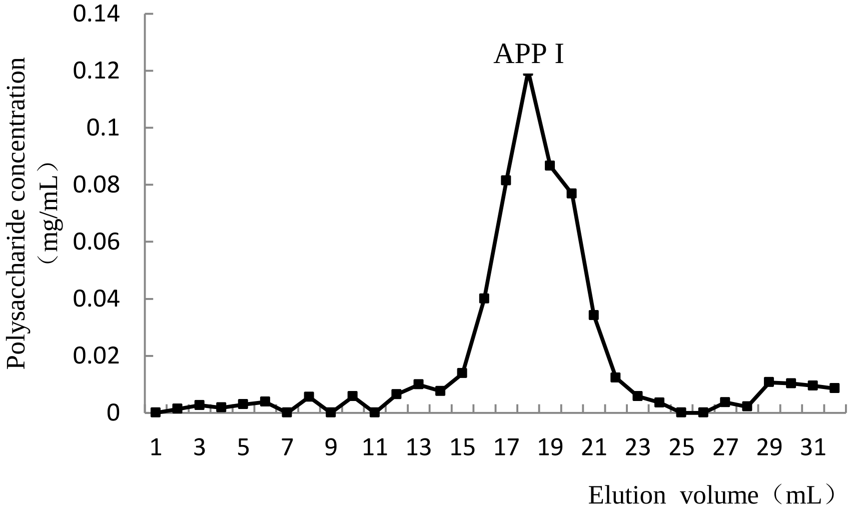

b

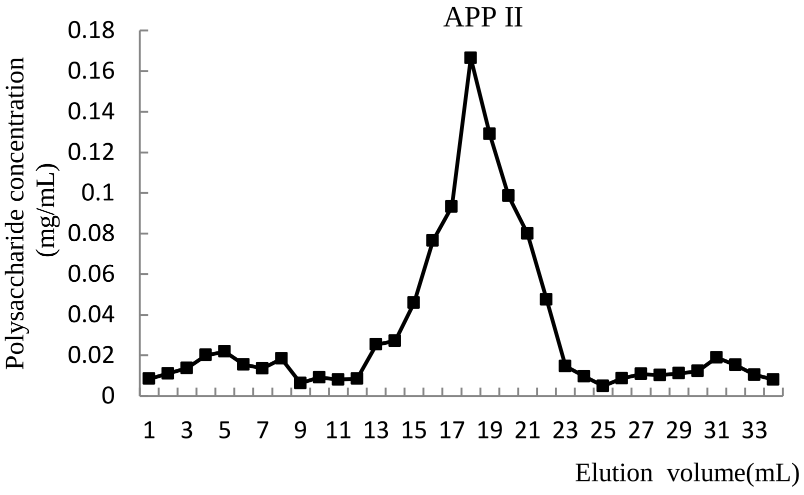

c

Supplementary Fig. S1. Purification of crude *A. polytricha* polysaccharide-peptides. (a) The crude APP was separated by chromatography on DEAE-cellulose column into 2 fractions: D1 and D2. (b) D1 was further subjected to Sephadex 200 column chromatography which generated a single symmetrical peak. (c) D2 was further separated by Sephadex 200 column chromatography which also generated a single symmetrical peak.

2. *Monosaccharide composition*

Monosaccharide composition analysis revealed mannose and xylose were the major sugars, while contain glucose and a small amount of arabinose and galactose (SIFig.2a, b). APPI was composed of Ara, Xyl, Man, Glc and Gal in a molar ratio of 1:38.3:46.2:15.4:4.4, while the corresponding molar ratio for APPII was 1:71.5:99.2:10:5.1.

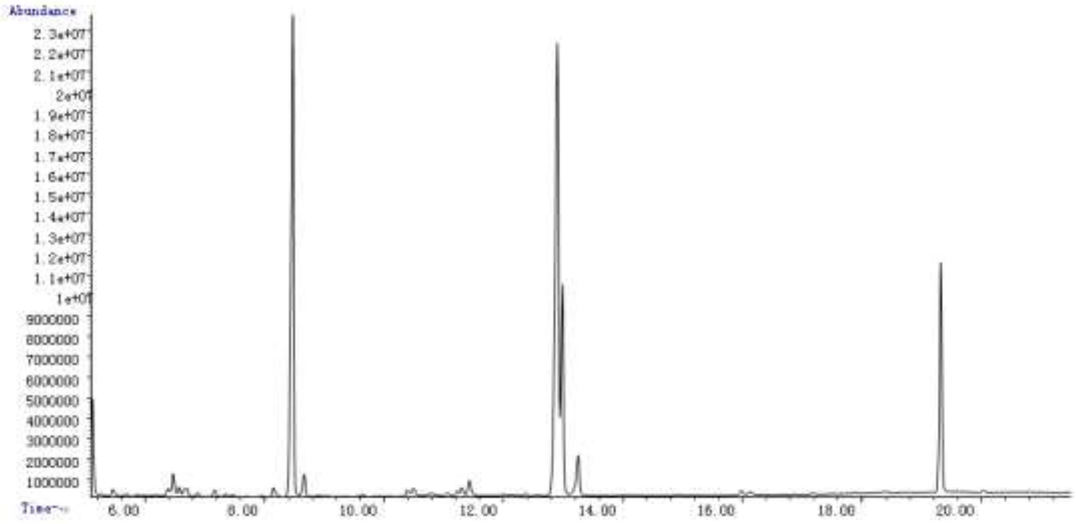

a

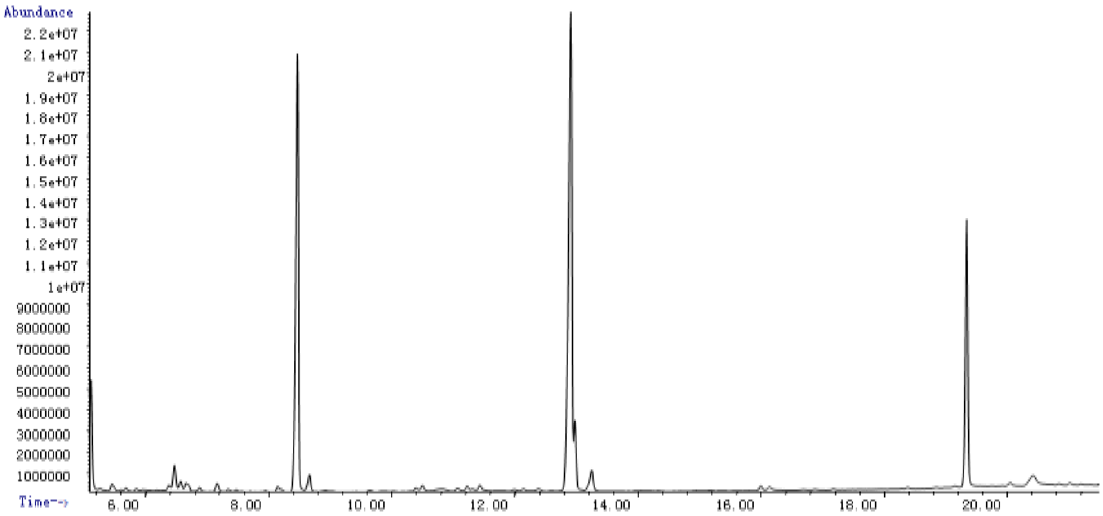

b

Supplementary Fig. S2. GC-MS chromatograms of *A. polytricha* polysaccharide-peptides. (a) APPI, (b) APPII

3. *Histological analysis of cellular lipid*

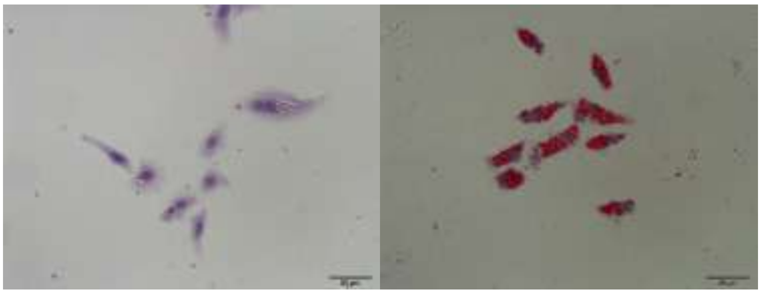

Control Model

Supplementary Fig. S3. Microscopic view of Oil-red O staining in HepG2 cells. (a) Control, (b) Model

4. *Fatty liver animal model foundation*

The biochemical analysis showed that HFD induce the levels of TG, TC and LDL-C significantly increasing, and elevate the levels of ALT and AST in serum. The HFD rats gained weight faster than those with SLD, at the ratio of 105.43%, and the liver weights in the model groups were heavier than that in control group. AST and ALT activities in the serum have been commonly used as biochemical markers for hepatic damage. The significantly elevated AST and ALT expression levels indicated the increased permeability and damage of hepatocytes.

Supplementary Table S1. Biochemical markers in HFD-model groups

	TG (mmol/L)	TC (mmol/L)	HDL-C (mmol/L)	LDL-C (mmol/L)	ALT (U/L)	AST (U/L)	Increasing ratio of Body weights(%)
Control group(SLD)	0.75±0.04	2.46±0.32	1.46±0.39	0.11±0.05	94.8±9.86	116.76±4.66	61.63
Model group(HFD)	1.51±0.45*	4.17±0.25*	1.14±0.39	1.32±0.53*	128.48±12.82*	260.17±18.70*	105.43*

Data are presented as means ± SEM (n = 6).*P < 0.05 compared with the control group
